# Supplementary material for: COZOID: contact zone identifier for visual analysis of protein-protein interactions
Source: BMC Bioinformatics. 2018 Apr 6;19:125. doi: 10.1186/s12859-018-2113-6 (PMC5889581; doi:10.1186/s12859-018-2113-6)
Supplement: Supplementary file 2 — Software build. Executable binary file of the software tool. (ZIP 118,784 kb) [file 12859_2018_2113_MOESM2_ESM.zip › cozoid/platform/docs/testng-6.5.1-javadoc.zip!/org/testng/CommandLineArgs.html]

CommandLineArgs (TestNG)


---


|  |  |  |
| --- | --- | --- |
| **Overview** \| **Package** \| **Class** \| **Tree** \| **Deprecated** \| **Index** \| **Help** | |  |
| **PREV CLASS**   **NEXT CLASS** | **FRAMES**    **NO FRAMES**     **All Classes** |  |
| SUMMARY: NESTED \| FIELD \| CONSTR \| METHOD | DETAIL: FIELD \| CONSTR \| METHOD |  |


---


## org.testng Class CommandLineArgs

```
java.lang.Object
  org.testng.CommandLineArgs
```

---

``` public class CommandLineArgs extends java.lang.Object ```

---

| **Field Summary** | |
| --- | --- |
| `java.util.List<java.lang.String>` | `commandLineMethods` |
| `static java.lang.String` | `CONFIG_FAILURE_POLICY` |
| `java.lang.String` | `configFailurePolicy` |
| `static java.lang.String` | `DATA_PROVIDER_THREAD_COUNT` |
| `java.lang.Integer` | `dataProviderThreadCount` |
| `java.lang.Boolean` | `debug` |
| `static java.lang.String` | `DEBUG` |
| `static java.lang.String` | `EXCLUDED_GROUPS` |
| `java.lang.String` | `excludedGroups` |
| `java.lang.String` | `groups` |
| `static java.lang.String` | `GROUPS` |
| `java.lang.String` | `host` |
| `static java.lang.String` | `HOST` |
| `java.lang.Boolean` | `junit` |
| `static java.lang.String` | `JUNIT` |
| `java.lang.String` | `listener` |
| `static java.lang.String` | `LISTENER` |
| `static java.lang.String` | `LOG` |
| `java.lang.String` | `master` |
| `static java.lang.String` | `MASTER` |
| `static java.lang.String` | `METHOD_SELECTORS` |
| `static java.lang.String` | `METHODS` |
| `java.lang.String` | `methodSelectors` |
| `java.lang.Boolean` | `mixed` |
| `static java.lang.String` | `MIXED` |
| `static java.lang.String` | `OBJECT_FACTORY` |
| `java.lang.String` | `objectFactory` |
| `static java.lang.String` | `OUTPUT_DIRECTORY` |
| `java.lang.String` | `outputDirectory` |
| `static java.lang.String` | `PARALLEL` |
| `java.lang.String` | `parallelMode` |
| `java.lang.Integer` | `port` |
| `static java.lang.String` | `PORT` |
| `static java.lang.String` | `RANDOMIZE_SUITES` |
| `java.lang.Boolean` | `randomizeSuites` |
| `java.lang.String` | `reporter` |
| `static java.lang.String` | `REPORTER` |
| `static java.lang.String` | `SKIP_FAILED_INVOCATION_COUNTS` |
| `java.lang.Boolean` | `skipFailedInvocationCounts` |
| `java.lang.String` | `slave` |
| `static java.lang.String` | `SLAVE` |
| `static java.lang.String` | `SUITE_NAME` |
| `static java.lang.String` | `SUITE_THREAD_POOL_SIZE` |
| `static java.lang.Integer` | `SUITE_THREAD_POOL_SIZE_DEFAULT` |
| `java.util.List<java.lang.String>` | `suiteFiles` |
| `java.lang.String` | `suiteName` |
| `java.lang.Integer` | `suiteThreadPoolSize` |
| `static java.lang.String` | `TEST_CLASS` |
| `static java.lang.String` | `TEST_JAR` |
| `static java.lang.String` | `TEST_NAME` |
| `static java.lang.String` | `TEST_NAMES` |
| `static java.lang.String` | `TEST_RUNNER_FACTORY` |
| `java.lang.String` | `testClass` |
| `java.lang.String` | `testJar` |
| `java.lang.String` | `testName` |
| `java.lang.String` | `testNames` |
| `java.lang.String` | `testRunnerFactory` |
| `static java.lang.String` | `THREAD_COUNT` |
| `java.lang.Integer` | `threadCount` |
| `static java.lang.String` | `USE_DEFAULT_LISTENERS` |
| `java.lang.String` | `useDefaultListeners` |
| `java.lang.Integer` | `verbose` |
| `static java.lang.String` | `VERBOSE` |
| `static java.lang.String` | `XML_PATH_IN_JAR` |
| `static java.lang.String` | `XML_PATH_IN_JAR_DEFAULT` |
| `java.lang.String` | `xmlPathInJar` |


**Constructor Summary**

`CommandLineArgs()`


**Method Summary**

**Methods inherited from class java.lang.Object**

`clone, equals, finalize, getClass, hashCode, notify, notifyAll, toString, wait, wait, wait`

**Field Detail**

### suiteFiles

```
public java.util.List<java.lang.String> suiteFiles
```

---


### LOG

```
public static final java.lang.String LOG
```

**See Also:**: Constant Field Values

---


### VERBOSE

```
public static final java.lang.String VERBOSE
```

**See Also:**: Constant Field Values

---


### verbose

```
public java.lang.Integer verbose
```

---


### GROUPS

```
public static final java.lang.String GROUPS
```

**See Also:**: Constant Field Values

---


### groups

```
public java.lang.String groups
```

---


### EXCLUDED\_GROUPS

```
public static final java.lang.String EXCLUDED_GROUPS
```

**See Also:**: Constant Field Values

---


### excludedGroups

```
public java.lang.String excludedGroups
```

---


### OUTPUT\_DIRECTORY

```
public static final java.lang.String OUTPUT_DIRECTORY
```

**See Also:**: Constant Field Values

---


### outputDirectory

```
public java.lang.String outputDirectory
```

---


### JUNIT

```
public static final java.lang.String JUNIT
```

**See Also:**: Constant Field Values

---


### junit

```
public java.lang.Boolean junit
```

---


### MIXED

```
public static final java.lang.String MIXED
```

**See Also:**: Constant Field Values

---


### mixed

```
public java.lang.Boolean mixed
```

---


### LISTENER

```
public static final java.lang.String LISTENER
```

**See Also:**: Constant Field Values

---


### listener

```
public java.lang.String listener
```

---


### METHOD\_SELECTORS

```
public static final java.lang.String METHOD_SELECTORS
```

**See Also:**: Constant Field Values

---


### methodSelectors

```
public java.lang.String methodSelectors
```

---


### OBJECT\_FACTORY

```
public static final java.lang.String OBJECT_FACTORY
```

**See Also:**: Constant Field Values

---


### objectFactory

```
public java.lang.String objectFactory
```

---


### PARALLEL

```
public static final java.lang.String PARALLEL
```

**See Also:**: Constant Field Values

---


### parallelMode

```
public java.lang.String parallelMode
```

---


### CONFIG\_FAILURE\_POLICY

```
public static final java.lang.String CONFIG_FAILURE_POLICY
```

**See Also:**: Constant Field Values

---


### configFailurePolicy

```
public java.lang.String configFailurePolicy
```

---


### THREAD\_COUNT

```
public static final java.lang.String THREAD_COUNT
```

**See Also:**: Constant Field Values

---


### threadCount

```
public java.lang.Integer threadCount
```

---


### DATA\_PROVIDER\_THREAD\_COUNT

```
public static final java.lang.String DATA_PROVIDER_THREAD_COUNT
```

**See Also:**: Constant Field Values

---


### dataProviderThreadCount

```
public java.lang.Integer dataProviderThreadCount
```

---


### SUITE\_NAME

```
public static final java.lang.String SUITE_NAME
```

**See Also:**: Constant Field Values

---


### suiteName

```
public java.lang.String suiteName
```

---


### TEST\_NAME

```
public static final java.lang.String TEST_NAME
```

**See Also:**: Constant Field Values

---


### testName

```
public java.lang.String testName
```

---


### REPORTER

```
public static final java.lang.String REPORTER
```

**See Also:**: Constant Field Values

---


### reporter

```
public java.lang.String reporter
```

---


### USE\_DEFAULT\_LISTENERS

```
public static final java.lang.String USE_DEFAULT_LISTENERS
```

**See Also:**: Constant Field Values

---


### useDefaultListeners

```
public java.lang.String useDefaultListeners
```

---


### SKIP\_FAILED\_INVOCATION\_COUNTS

```
public static final java.lang.String SKIP_FAILED_INVOCATION_COUNTS
```

**See Also:**: Constant Field Values

---


### skipFailedInvocationCounts

```
public java.lang.Boolean skipFailedInvocationCounts
```

---


### TEST\_CLASS

```
public static final java.lang.String TEST_CLASS
```

**See Also:**: Constant Field Values

---


### testClass

```
public java.lang.String testClass
```

---


### TEST\_NAMES

```
public static final java.lang.String TEST_NAMES
```

**See Also:**: Constant Field Values

---


### testNames

```
public java.lang.String testNames
```

---


### TEST\_JAR

```
public static final java.lang.String TEST_JAR
```

**See Also:**: Constant Field Values

---


### testJar

```
public java.lang.String testJar
```

---


### XML\_PATH\_IN\_JAR

```
public static final java.lang.String XML_PATH_IN_JAR
```

**See Also:**: Constant Field Values

---


### XML\_PATH\_IN\_JAR\_DEFAULT

```
public static final java.lang.String XML_PATH_IN_JAR_DEFAULT
```

**See Also:**: Constant Field Values

---


### xmlPathInJar

```
public java.lang.String xmlPathInJar
```

---


### TEST\_RUNNER\_FACTORY

```
public static final java.lang.String TEST_RUNNER_FACTORY
```

**See Also:**: Constant Field Values

---


### testRunnerFactory

```
public java.lang.String testRunnerFactory
```

---


### PORT

```
public static final java.lang.String PORT
```

**See Also:**: Constant Field Values

---


### port

```
public java.lang.Integer port
```

---


### HOST

```
public static final java.lang.String HOST
```

**See Also:**: Constant Field Values

---


### host

```
public java.lang.String host
```

---


### MASTER

```
public static final java.lang.String MASTER
```

**See Also:**: Constant Field Values

---


### master

```
public java.lang.String master
```

---


### SLAVE

```
public static final java.lang.String SLAVE
```

**See Also:**: Constant Field Values

---


### slave

```
public java.lang.String slave
```

---


### METHODS

```
public static final java.lang.String METHODS
```

**See Also:**: Constant Field Values

---


### commandLineMethods

```
public java.util.List<java.lang.String> commandLineMethods
```

---


### SUITE\_THREAD\_POOL\_SIZE

```
public static final java.lang.String SUITE_THREAD_POOL_SIZE
```

**See Also:**: Constant Field Values

---


### SUITE\_THREAD\_POOL\_SIZE\_DEFAULT

```
public static final java.lang.Integer SUITE_THREAD_POOL_SIZE_DEFAULT
```

---


### suiteThreadPoolSize

```
public java.lang.Integer suiteThreadPoolSize
```

---


### RANDOMIZE\_SUITES

```
public static final java.lang.String RANDOMIZE_SUITES
```

**See Also:**: Constant Field Values

---


### randomizeSuites

```
public java.lang.Boolean randomizeSuites
```

---


### DEBUG

```
public static final java.lang.String DEBUG
```

**See Also:**: Constant Field Values

---


### debug

```
public java.lang.Boolean debug
```


**Constructor Detail**

### CommandLineArgs

```
public CommandLineArgs()
```


---


|  |  |  |
| --- | --- | --- |
| **Overview** \| **Package** \| **Class** \| **Tree** \| **Deprecated** \| **Index** \| **Help** | |  |
| **PREV CLASS**   **NEXT CLASS** | **FRAMES**    **NO FRAMES**     **All Classes** |  |
| SUMMARY: NESTED \| FIELD \| CONSTR \| METHOD | DETAIL: FIELD \| CONSTR \| METHOD |  |


---
